# Processing Newborn Bloodspot Screening Results for CF

**DOI:** 10.3390/ijns6020025

**Published:** 2020-03-25

**Authors:** Jürg Barben, Jane Chudleigh

**Affiliations:** 1 Division of Paediatric Pulmonology & CF Centre, Children’s Hospital of Eastern Switzerland, 9006 St. Gallen, Switzerland; 2 School of Health Sciences, City, University of London, London EC1V 0HB, UK; j.chudleigh@city.ac.uk

**Keywords:** cystic fibrosis, newborn screening, presumptive diagnosis, sweat test, parental information

## Abstract

Every newborn bloodspot screening (NBS) result for cystic fibrosis (CF) consists of two parts: a screening part in the laboratory and a clinical part in a CF centre. When introducing an NBS programme, more attention is usually paid to the laboratory part, especially which algorithm is most suitable for the region or the country. However, the clinical part, how a positive screening result is processed, is often underestimated and can have great consequences for the affected child and their parents. A clear algorithm for the diagnostic part in CF centres is also important and influences the performance of a CF NBS programme. The processing of a positive screening result includes the initial information given to the parents, the invitation to the sweat test, what to do if a sweat test fails, information about the results of the sweat test, the inconclusive diagnosis and the carrier status, which is handled differently from country to country. The time until the definitive diagnosis and adequate information is given, is considered by the parents and the CF team as the most important factor. The communication of a positive NBS result is crucial. It is not a singular event but rather a process that includes ensuring the appropriate clinicians are aware of the result and that families are informed in the most efficient and effective manner to facilitate consistent and timely follow-up.

## 1. Introduction

Many countries have now introduced newborn bloodspot screening (NBS) for cystic fibrosis (CF) using a large variety of protocols [1,2]. After proper collection of the dried blood spot specimen, each NBS programme consists of a screening part in the laboratory and a diagnostic part in a CF centre. Most NBS programmes use a 2- or 3-step procedure in the laboratory, with the measurement of immunoreactive trypsinogen (IRT) being the first step [2]. The choice of the second step depends on the presence of CF transmembrane conductance regulator (CFTR) mutations, the health care system and the legislation of a country. Most programs today have CFTR determination as the second step; some use pancreatitis-associated protein (PAP) determination or a second IRT measurement. Therefore, the definition of a positive NBS screening result is handled slightly differently depending on the algorithm used. Like any other screening programme, a positive NBS result requires a confirmatory diagnosis, which for CF is done by means of a sweat test [3]. In accordance with WHO screening guidelines [4], the aim of every NBS programme is to detect children with CF as early as possible and to initiate the appropriate treatment. This means that the sweat test and a clinical evaluation should be performed as soon as possible in an experienced CF centre so that children with a confirmed CF diagnosis can be treated as early as possible. According to the current ECFS guidelines, infants with a confirmed diagnosis after NBS should be seen by the CF specialist team within one month (and no later than 58 days after birth) [3,5]. At the same time, false-positive screening results leading to recalls and sweat tests should be minimised as far as possible. The procedure following a positive NBS result (initial information to parents, invitation to the sweat test, information on the results of the sweat test, inconclusive diagnosis and carrier status) is different from country to country. For parents, the waiting period until the final diagnosis and information adapted to their knowledge is the most important part of the screening process [6]. In a survey of CF specialists, timeliness was also deemed very important by all respondents to ensure that children and their families were seen as soon as possible after receiving the initial positive NBS result [7].

## 2. Sweat Test—The Gold Standard Confirmation Test of a Positive NBS Result

**Figure 1:**
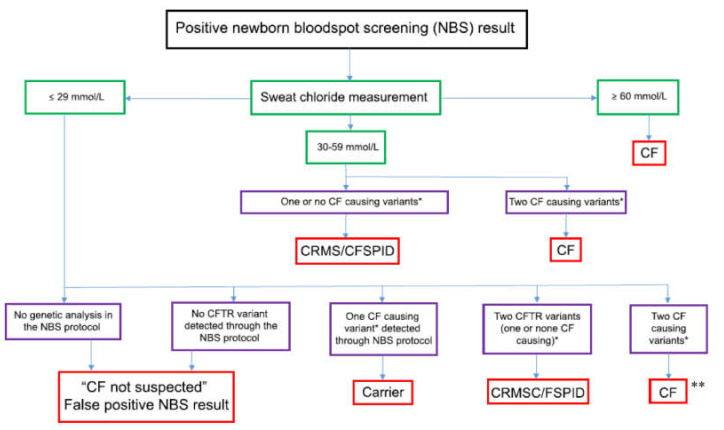
Adapted from reference [12] An algorithm for the designation of infants following a positive newborn bloodspot screening (NBS) result. (CF, cystic fibrosis; CFTR, CF transmembrane conductance regulator (gene); CRMS, CFTR-related metabolic syndrome; CFSPID, CF screen positive, inconclusive diagnosis; CRMS/CFSPID, harmonised definition). * Characterised by the CFTR2 website. ** If two CF causing mutations are present, a repetition of a sweat test is not necessary, but the parents should be genetically tested to exclude the presence of variants in the *cis* form.

It has been acknowledged that diagnosing CF is complex and that despite consensus guidelines being published on the diagnosis of CF, there is evidence that these are not applied consistently [3,5]. To confirm a positive NBS result, the determination of chloride concentration in the sweat is still considered the gold standard, because it investigates the function of the chloride channel, which is crucial in CF [8,9]. Most screened infants have little or no clinical manifestation of the disease, making sweat testing the main diagnostic tool to discriminate between children with and without CF [3,5,8,10]. A positive NBS result always requires a confirmatory sweat test, as there may be laboratory errors (mix-up of samples) or the two CFTR mutations detected in the genetic test are on the same allele [3,11]. The diagnostic algorithm of sweat testing is shown in Figure 1 [12].

However, sweat collection in infants is challenging and must be performed according to current guidelines [9,13,14]. A sufficient amount of sweat is needed to determine the chloride concentration; the smaller the children are or the lighter they are, the more difficult it is to obtain sufficient sweat. A sweat test should not be performed in children <2 kg, and the rates of sweat collections with insufficient volume (called Quantity Not Sufficient, QNS) are below 10% if the child weighs ≥4 kg [15,16]. Collecting sweat from two sites (left and right arm), as is recommended in the US, can reduce the QNS rate. The minimal standards and the diagnostic standards for laboratories performing sweat testing according to the recently revised ECFS best practice guidelines, are listed in Table 1 and Table 2 [5].

The recommendations of the US Cystic Fibrosis Foundation (CFF) say that the proportion of unsuccessful sweat tests in infants should be less than 10% [9,17]. In reality, the failure rate can be as high as 40% during the first three months of life. The rates of QNS samples is higher the earlier you test these children [15,17,18,19,20]. More effective NBS protocols have earlier results but higher QNS rates as babies are lighter at the time of testing.

Nowadays, the sweat collection is more frequently undertaken with a capillary tube system (Macroduct™), which was introduced in 1986 and only needs 15 μL of sweat to perform a chloride analysis [21,22,23,24]. Measuring conductivity using the Sweat-Check™ analyser has been suggested to be as effective as chloride determination in discriminating healthy children from those with CF [21,25,26,27]. Nanoduct™ is a newer sweat conductivity analysis system that was specially developed for newborns as it requires only 3–5 μL of sweat and measures conductivity in situ [28]. A few studies have assessed and confirmed its ability to discriminate between those affected by CF and healthy children [16,29,30,31,32,33]. According to current guidelines, conductivity measurement is an accepted method to rule out CF, but for a definite diagnosis, the determination of sweat chloride is required as it is better validated [9,14,24]. As the Nanoduct™ system does not require further laboratory equipment and personnel, it could have a role in remote areas where resources are limited and for ambulatory sweat testing.

## 3. Presumptive Diagnosis or How to Proceed if a Sweat Test Fails

**Figure 2:**
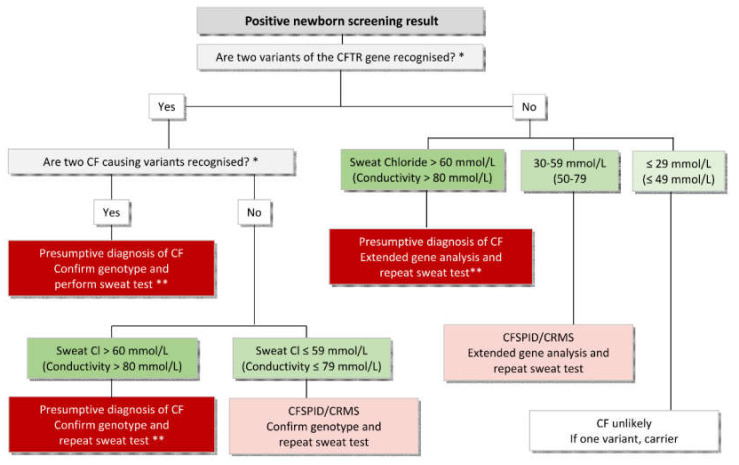
Diagnostic algorithm after a positive NBS result in the CF centre. * According to the CFTR2 website. ** Infants at this point have a presumptive diagnosis of CF and treatment should be established. Further testing is required to consolidate the diagnosis.

In some children, a determination of the sweat chlorides is not possible because of too little sweat production (QNS). So far, most algorithms do not explain what to do when sweat tests fail. Sometimes it is delayed until the child is older or heavier and this can take weeks before a sweat test is possible, and can lead to great uncertainty among parents [6]. In the Swiss NBS programme, if a sweat test was not possible, the sweat test was initially repeated after a few weeks or months until it was successful. However, this led to longer waiting times for definite diagnosis, and anxiety among families. After one year, the algorithm was changed, and genetic testing was directly performed after a sweat test failure. This led to greater detection of unclear cases, now called CF Screen Positive Inconclusive Diagnosis (CFSPID), but no reduction in waiting time. After that, the algorithm was again changed, and measurement of faecal elastase (FE) was introduced that significantly reduced the number of CFSPID infants being identified and improved the timeliness to start pancreatic enzyme replacement therapy (PERT) [34]. This well-documented change in diagnostic procedure highlights the fact that the algorithm for the diagnostic part of the newborn screening used in the CF centres is also important and affects the performance of a CF–NBS programme with regard to the ratio CF:CFSPID and the time until definite diagnosis. In the case of a high clinical probability of a CF diagnosis, one should not wait until the chloride result of a sweat test is available. A positive NBS for CF and a high sweat conductivity make the diagnosis of CF very likely and further investigations (determination of faecal elastase, confirm CFTR variants if necessary) should be initiated at first clinical visit. If symptoms such as lack of weight gain or steatorrhoea are present, start of PERT and salt supplements should not be delayed. A possible pathway is shown in Figure 2. In case of two CFTR variants and a negative sweat test the parents should be genetically tested to exclude the presence of variants in *cis* form. The definite confirmation of the diagnosis with a sweat chloride measurement should be performed at a later stage, as soon as the child reaches a higher weight (>4000g) [34]. This is also important because in the future, sweat chloride values will be a surrogate marker to determine the effect of CFTR modulators in early childhood.

## 4. Unintended Effects of Newborn Screening for CF

Depending on the algorithm chosen, NBS for CF results in recognition of unclear cases, now called CFSPID. Evaluation and management of these infants can be quite challenging [35,36]. This is especially the case if the second step in the screening algorithm is to search for a large number of CFTR mutations whose clinical relevance is different or not yet known. Depending on the chosen algorithm in the diagnostic process in a CF centre, there are also more and more unclear cases [34]. These children have in most cases a favourable prognosis and there is no evidence for improvement through early treatment [37]. In these unclear cases, the disadvantages of early detection with unnecessary medicalisation and burden to the family may outweigh the benefits of early detection [38].

False-positive results are a challenge to every screening programme, since they cause parental anxiety and unnecessary medical examinations [39,40]. NBS programmes using DNA analysis in their algorithm have improved specificity but identify heterozygote carriers, which can cause some anxiety and depression in affected families [6].

False negative results are also a challenge for screening programmes, as parents may feel reassured that the screening did not show a positive result and may consider that CF has been ruled out and possibly be less likely to go to the doctor later if the symptoms are present. Even in the best performing NBS programmes, some CF-affected individuals will be missed. The extent depends on the chosen cut-off value of the initial IRT measurement (false negatives in up to 8%) and the selected algorithm in the screening phase, and needs to be balanced with achieving a reasonable positive predicted value (PPV). Children with meconium ileus may have a negative NBS result, but always require an assessment with sweat and genetic testing.

## 5. Consequences for Other Family Members

Once a CF diagnosis has been confirmed (or ruled out), the parents and other family members may be offered genetic screening and counselling. All siblings of a positively screened child need to be screened for CF with a sweat test. Asymptomatic adult family members may wish to be screened for carrier status to allow them to make informed choices about reproduction planning and prenatal screening. Adequate genetic counselling should also be arranged or made available to them.

## 6. Communicating Positive NBS Results for CF 

Providing clear information to families is an important part of the NBS process to minimise unnecessary stress and anxiety. To date, there is no internationally accepted approach on how best to do this [7]. Communicating positive NBS results for any condition is not an event but a process that starts with the moment the result is identified as being above the agreed “cut off” and ends when the parents are given the definitive diagnosis for their child [41]. As a positive NBS result in itself is not diagnostic, and further tests are required to confirm or refute the NBS result before a definitive diagnosis is given to the family, this presents a period of uncertainty for families. This may be exacerbated in NBS for CF due to the range of possible outcomes, including a carrier result, confirmation of a diagnosis of CF or a designation of CFSPID, and the wide clinical spectrum that particularly the latter two presents.

Well-informed parents are less stressed by the screening process [6,39,40,42,43]. NBS programmes debate the antenatal information about the screened diseases in an NBS programme that should be given to new parents, who are already overloaded with information and brochures. In Switzerland, some parents felt that details about the screening test and the disease were missing when they were invited by phone for diagnostic evaluation. But information provided during this phone call is deliberately minimal without mentioning the respective disease, so as to reduce parental anxiety before they receive accurate information from a specialist [6].

When children with a positive NBS results are tested in a CF centre, their parents can feel anxious and depressed whilst awaiting definitive results [39,40]. CF specialists should be aware of the effect of the initial information given to parents and later in the CF centres, since this information can either reassure or cause anxiety to parents. The period between informing parents about a positive screening result and their appointment at the CF centre should be as short as possible. Parents should only be contacted when an appointment can be offered the following day.

According to the current ECFS best practice guidelines [5], a CF specialist should discuss a confirmed or inconclusive CF diagnosis in person with the parents (and not on the phone), and the family should receive written information to read after the consultation. Detailed information should also be sent to the family doctor or paediatrician. Families should be informed of further procedures and controls, the function and achievements of the CF research and made aware of opportunities for participation in clinical trials. In the case of a child identified to be a CF carrier, the family should receive a verbal report of the result, and written information should be sent to the family doctor or paediatrician. This information should be clear that (1) the infant does not have CF, (2) the baby is a healthy carrier of this mutation, (3) future pregnancies for the parents are not free of risk of CF and the parents may opt for genetic counselling, and (4) there are implications that could affect reproductive decision making for extended family members and the infant when they are of child-bearing age. A recently published US epidemiological study concluded that CF-related conditions, such as pancreatitis, infertility, diabetes, etc., were more prevalent among carriers than controls, whereas the individual-level risk remained low for most conditions [44]. This could have implications for future genetic counselling, but until these results are confirmed by other studies in other countries, CF carriers should not be medicalised unnecessarily.

A Swiss study exploring parents’ perspective of NBS for CF found that parental dissatisfaction with the communication of the NBS result was associated with poor information provision about the NBS result and the actual disease, again demonstrating the importance of ensuring the information is delivered by someone who is well-informed [6]. An international study with CF clinicians found that in the EU, Australia and New Zealand, a range of professionals are responsible for communicating the positive NBS to families, including midwives, the CF centre, the CF nurse, the NBS laboratory as well as the primary care physician, which had the potential to influence the quality of the information provided to the family [7].

Regardless of who is responsible for communicating the positive NBS result for CF, provision of complete and accurate information reduced parental anxiety and facilitated better decision making regarding seeking diagnostic testing and support from the CF team thereafter. Therefore, whoever is responsible for the initial communication with families should have condition-specific knowledge and be familiar with the NBS algorithm being used [7].

## 7. Feedback and Tracking

Monitoring the performance of NBS is important as it ensures compliance with relevant guidelines and a high standard of care [5]. This requires good data collection to assess the success of the screening. Many countries have already implemented formal mechanisms to ensure that children are seen in time and that specific protocols are followed, and they report their quality and compliance data [2]. However, as the protocols and associated reported variables differ, it is difficult to draw comparisons between programmes.

In some countries, the process of following up a positive NBS result is less straightforward, which poses difficulties and concerns for CF clinicians. Data from an international study with CF clinicians highlighted concerns in certain countries (particularly Turkey and Germany) regarding ensuring all children who received a positive NBS result received the appropriate diagnostic testing and were followed up in a timely manner by the CF team [7]. This was attributed to the mechanisms in place for communicating the positive NBS result for CF to the child’s family. In Germany, the NBS laboratory informs the birth clinic who then informs the parents about their child’s positive NBS result. In Turkey, processes were governed by who was seen to “own” the information about the positive NBS result, which in turn determined who was responsible for communicating the positive NBS result to the family. This was considered to have the potential to influence parental perceptions of the urgency and/or necessity for their child to be followed up by the CF team. In addition, in Turkey and Germany, parents are expected to arrange the sweat test after they have been informed of their child’s positive NBS result. In Germany, this was due to legal requirements that meant the CF centre could not be sent patient details. This raised concerns for CF clinicians in terms of ensuring that all babies who have a positive NBS result have a sweat test and are seen by the CF team in a timely manner.

## 8. Conclusions

Processing of a positive NBS screening result for CF has an important role in the success of a CF NBS programme, which is often underestimated when working out the optimal screening algorithm in the laboratory. It is a complex process not least because of the range of possible outcomes and the clinical uncertainty that this can present. Guidelines exist to assist clinicians but there is evidence these are used inconsistently for a range of reasons, including geographical, logistical, legal, financial and cultural constraints. Communication of positive NBS results is also challenging for many of the same reasons and presents a process rather than a singular event. However, whatever process is adopted must ensure this happens consistently and in a timely manner. Complete international harmonisation of NBS for CF is neither practical nor desirable but whatever processes are adopted must ensure outcomes for children, families and clinicians that are commensurate between programmes.

## Figures and Tables

**Table 1:** Minimal standards for laboratories performing sweat tests (according to the ECFS Guidelines, adapted from reference [5])

1	Sweat collection by experienced personnel (at least 150 sweat tests per annum) following national or international guidelines and subject to regular (at least annual) peer review.
2	Use of commercially available equipment approved for diagnostic use according to the national regulatory requirements or EU standards if no local ones are available.
3	Internal quality control (usually three samples) with acceptable limits of agreement for chloride before each sweat analysis.
4	Regular external quality assurance for the analyses according to national guidelines.
5	A high number of QNS (Quantity Not Sufficient) rates is a marker of technical issue. This necessitates renewing training for personnel experiencing sweat tests.

**Table 2:** Diagnostic standards of a sweat test (according to the ECFS Guidelines, adapted from reference [5])

1	The quantity of sweat should indicate an adequate rate of sweat production (15μL for Macroduct™ tube system).
2	The sweat sample should be processed immediately after sweat collection.
3	A sweat chloride value >59 mmol/L is consistent with a diagnosis of CF.
4	A sweat chloride value <30 mmol/L makes the diagnosis of CF unlikely. However, specific CF causing mutations can be associated with a sweat test below 30 mmol/L. These include c.3718-2477C N T (3849 + 10kbC N T) and mutations associated with varied clinical consequence, such as c.617T N G (L206W), c.1040G N A (R347H) and c.3454G N C (D1152H).
5	Individuals with sweat chloride values in the borderline range (30–59 mmol/L) should undergo a repeat sweat test and further evaluation in a specialist CF centre, including detailed clinical assessment and extensive CFTR gene mutation analysis.
